# Neuroinflammation in Neurodegenerative Disorders: Current Knowledge and Therapeutic Implications

**DOI:** 10.3390/ijms25073995

**Published:** 2024-04-03

**Authors:** Paras Mani Giri, Anurag Banerjee, Arpita Ghosal, Buddhadev Layek

**Affiliations:** Department of Pharmaceutical Sciences, School of Pharmacy, College of Health and Human Sciences, North Dakota State University, Fargo, ND 58105, USA; paras.giri@ndsu.edu (P.M.G.); anurag.banerjee@ndsu.edu (A.B.); arpita.ghosal@ndsu.edu (A.G.)

**Keywords:** Alzheimer’s disease, amyotrophic lateral sclerosis, anti-inflammatory drugs, Huntington’s disease, neurodegenerative disorders, Parkinson’s disease

## Abstract

Neurodegenerative disorders (NDs) have become increasingly common during the past three decades. Approximately 15% of the total population of the world is affected by some form of NDs, resulting in physical and cognitive disability. The most common NDs include Alzheimer’s disease, Parkinson’s disease, amyotrophic lateral sclerosis, and Huntington’s disease. Although NDs are caused by a complex interaction of genetic, environmental, and lifestyle variables, neuroinflammation is known to be associated with all NDs, often leading to permanent damage to neurons of the central nervous system. Furthermore, numerous emerging pieces of evidence have demonstrated that inflammation not only supports the progression of NDs but can also serve as an initiator. Hence, various medicines capable of preventing or reducing neuroinflammation have been investigated as ND treatments. While anti-inflammatory medicine has shown promising benefits in several preclinical models, clinical outcomes are often questionable. In this review, we discuss various NDs with their current treatment strategies, the role of neuroinflammation in the pathophysiology of NDs, and the use of anti-inflammatory agents as a potential therapeutic option.

## 1. Introduction

The term “neurodegenerative disorders (NDs)” refers to a broad category of illnesses marked by the gradual degradation of neurons, leading to cognitive deterioration, motor dysfunction, and other neurological symptoms [1,2]. Alzheimer’s disease (AD), Parkinson’s disease (PD), Huntington’s disease (HD), and amyotrophic lateral sclerosis (ALS), are among the many disorders that fall under the umbrella of neurodegenerative diseases [3]. However, these NDs are characterized by their distinct clinical appearances, afflicted brain areas, and underlying pathogenic mechanisms. For instance, AD is characterized by memory loss and cognitive decline, associated with beta-amyloid plaques and tau tangles. PD involves tremors, bradykinesia, and stiffness due to dopamine-producing neuron degeneration. HD manifests as involuntary movements and cognitive decline arising from CAG trinucleotide repeat expansion in the *HTT* gene. ALS leads to progressive muscle weakness and paralysis due to motor neuron degeneration.

NDs are the primary cause of physical and cognitive disability, affecting over 15% of the global population [4]. Over the last three decades, the number of patients has increased dramatically. Furthermore, the prevalence of chronic NDs is predicted to at least double over the next two decades. The two most common neurological diseases are AD and PD. In 2023, an estimated 6.7 million Americans 65 years of age and older have Alzheimer’s disease. If medical advancements are not made to prevent, stop, or cure AD, this figure may rise to 13.8 million by 2060 [5]. These numbers are anticipated to increase quickly as the American population ages. By 2040, it is predicted that the number of PD diagnoses in the United States will have doubled [6]. According to the Parkinson’s Foundation, around one million Americans live with PD. A 2022 Parkinson’s Foundation-sponsored research estimated almost 90,000 persons are diagnosed with PD in the United States each year. This indicates a 50% increase above the previously expected rate of 60,000 diagnoses yearly. Furthermore, between 1990 and 2015, the number of persons dying from neurological disorders increased by 36.7%, with stroke and infectious neurological diseases accounting for the majority of this rise [7].

The impact of NDs on people, families, and society is profound. A variety of physical, cognitive, and psychological symptoms are brought on by the progressive degradation and dysfunction of the neurological system that characterizes these disorders. In the case of individuals suffering from NDs, there is often a gradual loss of motor function, cognitive abilities, and/or sensory functions, depending on the specific disorder [8]. Thus, symptoms of these diseases can significantly impact an individual’s quality of life, affecting daily activities, independence, and overall well-being [9]. Anxiety, depression, and a feeling of helplessness are among the emotional difficulties that can arise while adjusting to the progressive nature of neurodegenerative diseases [10]. Significant healthcare expenses are associated with managing NDs, including appointments with physicians, prescription drugs, and, in certain situations, long-term care facilities [11]. NDs are predicted to become more common as the world’s population ages, presenting serious problems for public health systems everywhere. Finally, the economic burden of NDs, including healthcare costs and lost productivity, has wide-ranging implications for societies and economies.

## 2. General Causes of NDs

NDs, encompassing conditions like AD, PD, HD, and ALS, arise from a complex interplay of genetic, environmental, and lifestyle factors. Mutations in particular genes, such as the *APP*, *PSEN1*, and *PSEN2* genes associated with AD [12] and *HTT* in Huntington’s disease, contribute to familial variants of these NDs [13], and genetic factors play a major role in these cases. Numerous NDs are characterized by protein misfolding and aggregation, which involves the abnormal buildup of certain proteins such as tau, alpha-synuclein, and beta-amyloid. Environmental variables, such as extended exposure to toxic chemicals like pesticides, may increase susceptibility to NDs [14]. Damage to neurons is also caused by oxidative stress, characterized by an imbalance in reactive oxygen species and malfunctioning mitochondria [15].

Neuroinflammation is acknowledged to be a significant factor in the progression of neurological disorders, especially chronic NDs [16]. Chronic inflammation in the brain can contribute to the deterioration of neurons and intensify the symptoms associated with these conditions. There are many causes of neurodegeneration, but inflammation is a common attribute among all of them. More detailed discussions on neuroinflammation and its effects are examined thoroughly later in this review. Age is a significant risk factor since the incidence of many NDs increases with age. Researchers are investigating potential links between viral infections and NDs, because previous research has connected particular viruses to an increased risk of these conditions [17].

Lifestyle variables also play an important role in the progression of NDs. Unhealthy behaviors such as high-sugar and high-fat diets and alcohol and tobacco use have a deleterious impact on neurodegeneration. However, several components in our diets, like polyunsaturated fatty acids (e.g., docosahexaenoic acid and eicosapentaenoic acid), antioxidants (curcumin, resveratrol, carotenes, blueberry polyphenols, salvianolic acid A, sulforaphane, and vitamin C), as well as caloric restriction and physical activity may assist us in living better and longer lives [2,18,19]. Considering all these facts, developing successful preventive and therapeutic methods against NDs requires a thorough grasp of these complicated causes [18]. 

## 3. Common NDs and Their Treatment Approaches

In the following sections, we will briefly cover some common NDs and their current treatment strategies.

### 3.1. Alzheimer’s Disease (AD) 

**Figure 1:**
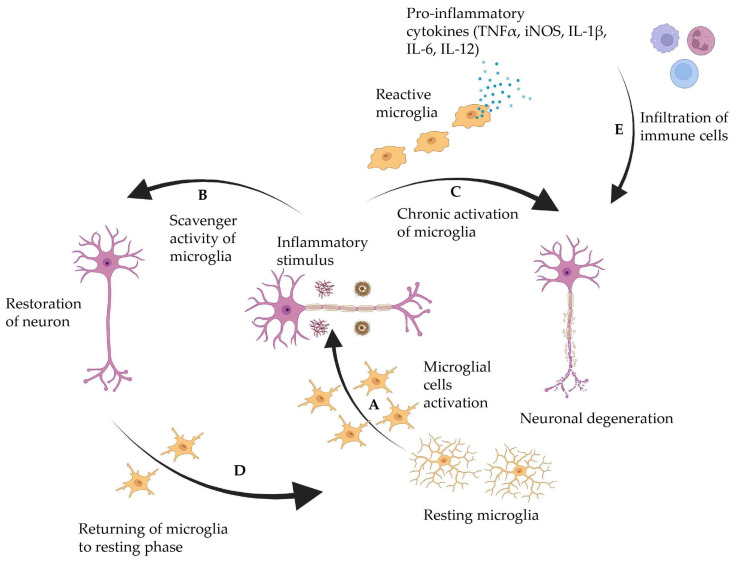
Role of microglia in AD progression. (A) Inactive microglia become activated by stimuli like amyloid β or tau protein deposition. (B) Complete clearance of amyloid β leads to restoration of neurons, and (D) microglia returns to the resting phase. In contrast, (C) failure in amyloid β clearance leads to chronic microglia activation that releases proinflammatory cytokines and neurotoxicity. (E) Entry of the peripheral immune system leads to neurodegeneration. This figure was created using Biorender.com.

AD is a brain disorder that gradually impairs memory, thinking abilities, and the capacity to perform even the most basic tasks [20]. The main pathophysiological hallmarks of AD are extracellular plaque deposition of amyloid beta (Aβ) peptide and intracellular neurofibrillary tangles of the microtubule-binding protein tau (τ) [21]. Amyloid-beta precursor protein (APP) is an integral membrane protein found in numerous tissues, but it is mainly concentrated at neuron synapses. APP is involved in various biological processes, including neuronal development, signaling, intracellular transport, and neuronal homeostasis. The altered cleavage of APP by β-secretases (BACE1) and γ-secretases secretes insoluble Aβ fibrils. The accumulation of Aβ fibrils hinders synaptic signaling. The further condensation of fibril manifests into plaque deposition, causing kinase activation. The activated kinase leads to hyperphosphorylation and the condensation of τ protein into polymerization of neurofibrillary tangles. Following the plaque deposition and neurofibrillary tangles, aggregation promotes microglial activation, promoting inflammatory cytokines and neurotoxicity (Figure 1) [22,23,24,25].

The approved medication for AD includes cholinesterase inhibitors, N-methyl-D-aspartate receptor antagonists (NMDA), antipsychotics, and disease-modifying immunotherapy [26]. Cholinesterase inhibitor drugs like donepezil, rivastigmine, or galantamine promote acetylcholine levels in the brain by inhibiting the acetylcholinesterase enzyme. The elevated level of acetylcholine promotes cholinergic neurotransmission and subdues cognitive impairment [27]. Memantine, a partial NMDA receptor antagonist, has been approved for moderate to severe AD. Overactivation of NMDA receptors has been observed in AD patients, leading to a higher level of glutamate in the brain [28]. The elevated glutamate levels result in neurotoxicity. Thus, NMDA receptor antagonists subdue the glutamatergic system and ameliorate cognitive and memory functions [29]. Brexpiprazole is the only FDA-approved atypical antipsychotic drug for AD patients for the management of agitation associated with dementia [30]. Symptomatic improvement by brexpiprazole is considered to result from antagonistic action in noradrenergic α1B and α2C and serotonergic 5-HT2A receptors and partial agonistic activity at 5-HT1A and dopaminergic D2 receptors [31,32]. Lecanemab and Aducanumab are monoclonal antibodies approved for AD treatments [26,33]. These therapies act by removing abnormal Aβ from the brain [34,35,36]. 

### 3.2. Parkinson’s Disease (PD)

PD is a progressive disorder caused by neuronal degeneration in the substantia nigra region of the brain, which regulates the body’s movement [37]. Usually, these neurons synthesize dopamine, a key brain neurotransmitter. Killing or impairing these neurons causes lower dopamine production, resulting in the movement issues associated with the condition. It has been shown that patients who have lost 80 percent or more of the dopamine-generating cells in the substantia nigra segment of their brains experience movement disorders such as tremors, muscle stiffness, bradykinesia, and postural instability [38,39,40].

The commonly used drugs for PD include dopamine precursors, dopamine agonists, monoamine oxidase-B (MAO-B) inhibitors, catechol-O-methyl transferase (COMT) inhibitors, anticholinergics, adenosine 2A antagonists, and N-methyl-D-aspartate (NMDA) antagonist. Levodopa, a dopamine precursor, is often considered the first-line drug for the treatment of PD. Following oral administration, it is quickly absorbed into the blood from the small intestine and enters the brain across the blood-brain barrier. Levodopa is decarboxylated to dopamine in the brain, particularly in the presynaptic terminals of dopaminergic neurons in the stratum. The presence of dopamine in the brain activates the dopamine receptors that aid in improving Parkinson’s symptoms. Levodopa is almost always given with carbidopa, which prevents the peripheral decarboxylation of levodopa before its delivery to the brain. This combination lowers the dose of levodopa and reduces or eliminates its gastrointestinal side effects [41].

Dopamine agonists such as pramipexole, ropinirole, apomorphine, or rotigotine directly activate dopamine receptors without having to be converted [42]. The duration of effect for clinically used dopamine agonists is significantly longer than that of levodopa. The majority of the oxidative metabolism of dopamine in the brain is carried out by monoamine oxidase-B. The MAO-B inhibitors selegiline, rasagiline, and safinamide enhance the availability of dopamine in the brain [43]. MAO-B inhibitors may be beneficial as monotherapy or combined with other drugs, such as levodopa. COMT inhibitors such as entacapone, tolcapone, and opicapone enhance dopamine concentration in the brain by inhibiting the peripheral degradation of levodopa [44,45]. Therefore, COMT inhibitors are often combined with levodopa to extend its duration of effect. The anticholinergic class of drugs minimizes tremors and muscle rigidity by blocking acetylcholine. Istradefylline is an adenosine receptor A2A antagonist that is well tolerated and effective in reducing daily “off” time, making it a valuable choice for individuals with severe PD. Amantadine is primarily used to help in reducing involuntary movements.

### 3.3. Amyotrophic Lateral Sclerosis (ALS)

ALS, also known as Lou Gehrig’s disease, is a rapidly progressive ND characterized by losing the motor neurons in the primary motor cortex, brainstem, corticospinal tracts, and spinal cord that regulate voluntary muscle movement and breathing [46]. As the motor neurons gradually deteriorate or die, they stop transmitting messages to the muscles, causing them to weaken, twitch, and become wasted (atrophy) over time. Eventually, ALS patients lose their capacity to initiate and control voluntary movements such as walking, talking, chewing, and breathing. ALS is a progressive disease, which means the symptoms worsen with time. Patients with ALS may experience other overlapping symptoms, including behavioral and cognitive abnormalities or even frontotemporal dementia, which can result in a highly varied clinical manifestation [47,48]. Most ALS patients die from respiratory failure and pneumonia within two to three years of onset. The exact cause of ALS is still unclear. About 5–10% of all ALS cases are inherited. The most often-identified genetic causes of familial ALS are the presence of a hexanucleotide repeat expansion in the *C9ORF72* gene and mutations in *SOD1*, *TARDBP*, and *FUS* genes [49]. These four genes account for over two-thirds of familial ALS cases [50]. 

The current standard of therapy for ALS is multimodal symptom management, which includes dietary and respiratory assistance. Riluzole is the most commonly prescribed medication for ALS, which primarily acts by inhibiting glutamate release [51]. It was first authorized in 1995 as an oral tablet used once a day at a dose of 100 mg. Although riluzole is typically safe and well tolerated in clinical practice, its effectiveness in ALS is limited, extending tracheostomy-free life by only 2–3 months [52]. The free radical scavenger edaravone, which helps slow the course of the disease, was licensed in 2017 as an intravenous infusion at a dose of 60 mg/day [53]. Even though the exact mechanism of action of the beneficial properties of edaravone against ALS is unknown, its therapeutic effects are deemed to be due to its antioxidant properties. An oral suspension of edaravone was approved in 2022. 

Tofersen, a new antisense oligonucleotide drug, was approved by the United States FDA in April 2023 under the Accelerated Approval Program for treating ALS patients with a superoxide dismutase (*SOD1*) gene mutation [54]. It is designed to selectively target the RNA generated by mutant *SOD1* genes, thereby preventing harmful SOD1 proteins from accumulating in the cerebrospinal fluid (CSF). Tofersen is administered by a lumbar puncture. A phase 1/2 clinical trial of 50 participants found that tofersen therapy was usually safe and reduced SOD1 protein levels in CSF. Relyvrio (AMX0035), a combination of sodium phenylbutyrate and taurursodiol, received FDA approval in September 2022 to treat ALS patients. It slows the progression of the disease by delaying or preventing the death of motor neuron cells.

### 3.4. Huntington’s Disease (HD)

HD is an autosomal dominant progressive ND that affects movement, emotions, and cognitive function. It is characterized by increased CAG trinucleotide repeats on the short arm of chromosome 4 in the huntingtin gene (*HTT*) [55]. The higher the number of CAG repeats in the *HTT* gene, the greater the likelihood of HD onset. Normal individuals have CAG repeat lengths ranging from 9 to 34 triplets, with a typical repeat length of 19 on normal chromosomes. People who have more than 36 CAG repeats are at a higher risk of developing HD, and patients who have more than 40 repetitions are destined to have the disease at some point in their lives [55,56,57]. 

The *HTT* gene that carries more than 36 CAG repeats leads to the synthesis of mutant *HTT*(mHTT) protein. These amino-terminal mHTT fragments lead to the formation of misfolded proteins, which then form aggregates in the nerve cells [58,59]. These aggregates eventually cause the death of nerve cells through various mechanisms, such as increased sensitivity to excitotoxicity like excess glutamate toxicity [60], mitochondrial dysfunction [61], caspase activation [62], and transcriptional dysregulation [63]. The usual age for HD onset is 40 years, but patients who suffer from juvenile HD start showing symptoms of bradykinesia and rigidity as early as 20 years of age [64]. 

The principal location of neuron loss in HD is the striatal region of the basal ganglia, leading to motor, cognitive, and behavioral dysfunction. The primary motor symptom of HD is chorea. Other characteristics are gait disturbances, dystonia, tics, problems initiating voluntary movements, saccadic eye movements, myoclonus, and dysarthria. In addition to motor dysfunction, HD also causes substantial behavioral and cognitive symptoms like dementia, depression, psychosis, apathy, impaired executive function, aggression, irritability, and personality changes [60]. 

To date, no specific drugs are available to cure or delay the progression of HD, but several drugs are used to provide symptomatic relief from some of the HD-associated complications [65]. The most often recommended HD drugs are intended to decrease chorea. Tetrabenazine and deutetrabenazine are medications that help treat HD-associated chorea. Tetrabenazine works mainly as a reversible, high-affinity inhibitor of monoamine uptake in presynaptic vesicles by preferentially binding to vesicular monoamine transporter 2 (VMAT-2) [66]. This inhibition enhances monoamine breakdown in the neurons, causing monoamine depletion, particularly dopamine [67]. Tetrabenazine has been reported to inhibit dopamine D2 receptors also. However, this affinity is 1000 times lower than its affinity for VMAT-2 [68,69]. This mechanism is unlikely to be responsible for its therapeutic benefits, although it might be implicated in uncommon acute dystonic responses. 

Deutetrabenazine is another drug approved to treat HD-associated chorea. It is an isotopic isomer of tetrabenazine in which deuterium atoms have replaced six hydrogen atoms. The presence of deuterium atoms increases its half-life, allowing for less frequent administration [70]. Both tetrabenazine and deutetrabenazine come with warnings concerning depression and suicidal tendencies [71]. Individuals with HD who used DBZ showed a considerably decreased probability of having neuropsychiatric adverse effects (agitation, depression, sleepiness, sleeplessness, and parkinsonism) compared to those who took tetrabenazine [72].

Apart from these drugs, other medications are used to tackle the symptoms. Antipsychotic medications such as olanzapine and risperidone may alleviate chorea and assist in controlling hallucinations, delusions, and violent outbursts. Some antipsychotic drugs have adverse effects that exacerbate HD’s muscular contraction symptoms. Therefore, HD patients who are taking antipsychotic medicines should be continuously monitored for potential adverse effects [73]. Olanzapine is an atypical antipsychotic agent that inhibits dopamine (D1, D2, and D4), histamine, serotonin (5-HT2A, 5-HT2C), and α-1 adrenergic receptors [74]. Like olanzapine, risperidone is also an atypical antipsychotic agent that selectively inhibits dopamine-D2 and serotonin-S2 (5-HT) receptors. Blocking both the D2 and 5-HT receptors is expected to reduce the hyperactive nerve transmission that produces tremors and improve depression in Huntington’s patients. 

Approximately 40% of HD patients suffer from partial or continuous depression, and one in every ten patients attempts suicide after being diagnosed with the condition [64,75]. HD-associated depression can be effectively treated with standard antidepressant drugs such as fluoxetine, sertraline, and citalopram. HD patients often exhibit behavioral symptoms such as OCD, aggressiveness, and bipolar illness. Patients suffering from these mood disorders can be treated with a variety of anticonvulsants, including lamotrigine and carbamazepine. 

The United States Food and Drug Administration has approved several medications for the management or symptomatic treatment of common NDs, as shown in Table 1.

## 4. Drugs Targeting Neuroinflammation in NDs

### 4.1. AD 

Neuroinflammation is considered to be closely associated with the development and progression of AD [91,92]. However, researchers are still trying to find out whether inflammation causes AD or vice-versa. Inflammation is a repair process in the body that normalizes in a short period. However, prolonged inflammation in the brain causes deleterious effects on nerve cells via the release of cytotoxic agents. Microglia and astrocytes are the primary sources for producing the various inflammatory cytokines that contribute to neuroinflammation [93]. Initially, microglial cell activation caused by Aβ deposition is considered a beneficial response for reducing the burden through phagocytosis [94,95]. However, prolonged microglial cell activation reduces their ability to bind and phagocytose Aβ. Additionally, sustained microglia activation constantly releases numerous proinflammatory cytokines, reactive oxygen species, and nitric oxide, leading to chronic inflammation that further deteriorates AD conditions [96]. 

Furthermore, epidemiological, neuroimaging, preclinical, and genetic evidence suggests that neuroinflammation is a key etiological feature in AD [96,97]. Therefore, multiple investigations have been conducted to find the potential use of anti-inflammatory agents for AD management. Even though epidemiological findings suggest the beneficial effects of non-steroidal anti-inflammatory drugs (NSAIDs) on AD, clinical trials have failed to obtain such a correlation. Such contrasting findings might be due to the incorrect NSAIDs being tested in clinical trials or the epidemiological findings being caused by some undetermined factors. Thus, a properly designed study is required to determine the effects of NSAIDs on AD. A recent study was performed using a logistic regression model to evaluate the prevalence and cognitive decline in AD patients taking various NSAIDs [98]. The result suggested that multiple NSAIDs are related to the low prevalence of AD but do not affect cognitive function. However, paracetamol (non-NSAID) also leads to a low prevalence of AD, implying that the connection of NSAIDs to low AD prevalence is due to a spurious correlation. On the other hand, diclofenac use led to both low prevalence and a reduced rate of cognitive decline [98].

**Figure 2:**
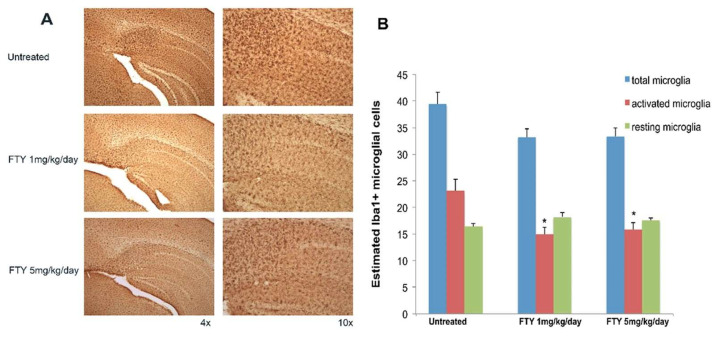
(**A**) Sections of hippocampus and subiculum immunostained for ionized calcium binding adaptor molecule 1 (Iba1) in 5xFAD mice untreated, treated with 1 mg/kg/day, and treated with 5 mg/kg/day of fingolimod. Untreated 3 month old 5xFAD mice showed significant increase in the number of activated microglial cells. (**B**) Quantitation of total, active, and resting variants of microglia in the hippocampus of 5xFAD untreated and fingolimod-treated groups. The number of activated Iba1-positive microglia significantly decreased in the hippocampus of 1 mg/kg/day, and 5 mg/kg/day fingolimod-treated groups compared with untreated group (* *p* < 0.05), (*n* = 8–10 mice/group) This figure is reprinted under the terms of the Creative Commons Attribution 4.0 International License from reference [99].

Despite the lack of concrete evidence, multiple preclinical studies have been conducted to assess the potential role of anti-inflammatory agents in AD management. Fingolimod is an S1P1 receptor antagonist prescribed for multiple sclerosis treatment. A study was conducted to determine if the anti-inflammatory activity of fingolimod provides neuroprotection in an AD mouse model [99]. Mice were treated with two doses of fingolimod (1 and 5 mg/kg/day) for two months, and various Aβ pathology, inflammatory, and neurochemical indicators were measured. Fingolimod was observed to lower both soluble and insoluble Aβ as determined by ELISA. Additionally, glial fibrillary acidic protein (GFAP) staining and the quantity of activated microglia were reduced by fingolimod (Figure 2).

Anti-inflammatory medications, such as ibuprofen, lower the risk of AD when taken before the onset of cognitive impairment. The exact mechanism responsible for this protection is as yet unknown. Therefore, a mechanistic study was conducted in APP–PS1 mice to unravel the neuroprotective role of ibuprofen [100]. Treating APP–PS1 mice with ibuprofen prevented cognitive decline without affecting glial inflammation or Aβ accumulation. As an alternative, ibuprofen may have altered hippocampus gene expression in neuronal plasticity pathways, resulting in higher levels of norepinephrine and dopamine. The gene most significantly downregulated by ibuprofen treatment was neuronal tryptophan 2,3-dioxygenase (TDO2), which encodes an enzyme that converts tryptophan to kynurenine. Neuronal COX-2 activity elevated the production of TDO2, and hippocampus TDO2 overexpression resulted in behavioral impairments. Similarly, Paul et al. [101] conducted a study using multiple NSAIDS and antihypertensive drugs in different murine models of AD. It was observed that NSAIDs—dexketoprofen and etodolac and the antihypertensive drugs penbutolol and Bendroflumethiazide—were able to reverse the cognitive impairment in AD mice models and lessen Aβ deposition in the hippocampus. 

### 4.2. PD

Multiple studies suggest that an increased inflammatory response plays a vital role in the pathogenesis of PD. In 1988, McGeer and colleagues conducted a post-mortem investigation in which they found that activated microglia were present in the substantia nigra of PD patients [102]. This finding established the connection between inflammation and PD for the first time. Since then, numerous clinical studies have shown higher levels of proinflammatory cytokines and microglial activation in post-mortem brains and CSF of PD patients in comparison with non-PD individuals [103,104,105]. These findings were further supported by case studies demonstrating that early-life brain injuries led to an increased incidence of PD in later life [106]. 

**Figure 3:**
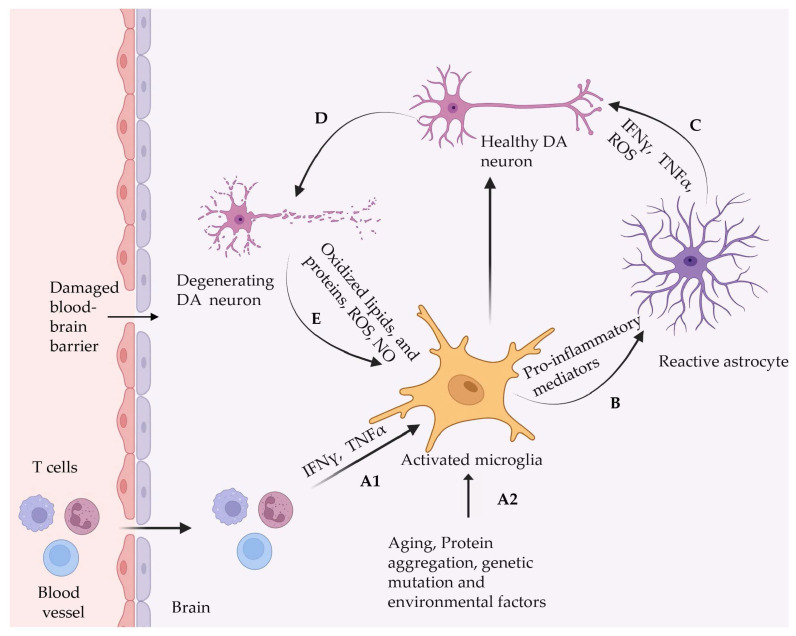
Role of inflammation in PD pathogenesis. (A1 and A2): Pathological conditions like genetic mutation, protein aggregation, aging, and cytokine released from infiltrated T cells activate microglia. (B) The release of inflammatory mediators from microglia activates astrocytes. (C) Release of IL-B, TNFα, and ROS from activated astrocytes causes dopaminergic neuron degeneration. (D) Degeneration of dopaminergic neurons due to chemokine release from astrocytes. (E) The chemicals released from degenerating neurons activate glial cells and aggravate inflammatory responses. This figure is adapted under the terms of the Creative Commons Attribution 4.0 International Li-cense from reference [111].

Similarly, patients suffering from allergic rhinitis, a condition with nasal airway inflammation, are at an increased risk of PD development [107]. The preclinical model also suggests inflammation is a major cause of deterioration of the nigrostriatal dopaminergic system [108]. Mice injected with bacterial lipopolysaccharide developed a provoked systemic immune response followed by degeneration of their substantia nigral dopaminergic neurons and the pathogenesis of PD [109]. Furthermore, Gao et al. [110] demonstrated that oxidative stress caused by microglia is linked to neuroinflammation and α-synuclein pathogenesis in chronic PD progression. Thus, from all this compelling evidence, it can be inferred that inflammation plays a vital role in chronic PD manifestations. Figure 3 depicts the role of neuroinflammation in the pathogenesis of PD.

Several studies have demonstrated the preventative role of many anti-inflammatory therapies, including ibuprofen [112], dexamethasone [113], minocycline [114], IL-10 [115], and pituitary adenylate cyclase-activating polypeptide [116] on dopaminergic cell death. However, the beneficial effects of anti-inflammatory medications on Parkinson’s patients are conflicting. One meta-analysis indicated that NSAIDs may not reduce the incidence of PD, with only ibuprofen appearing to have a slight protective effect [117]. In contrast, another meta-analysis found that with the exception of aspirin, NSAIDs may reduce the incidence of PD [118]. Minocycline, which exhibited neuroprotective efficacy in multiple in vitro and in vivo investigations, was ineffective in changing the course of early PD over 12 and 18 months in a randomized clinical study [119,120]. 

Ursolic acid, a natural pentacyclic triterpenoid carboxylic acid, is present in numerous fruits (e.g., apples, blueberries, cranberries), herbs (e.g., basil, peppermint, rosemary, thyme), and flowers. It is known to have potent anti-inflammatory and antioxidant activities. In a recent study, the anti-inflammatory potential of ursolic acid was studied in a 1-methyl-4-phenyl-1,2,3,6-tetrahydropyridine (MPTP)-intoxicated Parkinsonian mouse model [121]. Oral administration of ursolic acid (25mg/kg body weight) in PD model mice led to a significant decrease in various inflammatory parameters like ionized calcium binding adaptor molecule 1 (Iba1), nuclear transcription factor-κB (NF-κB), and tumor necrosis factor-alpha (TNF-α) in the substantia nigra pars compacta (SNpc) of MPTP-intoxicated animals. Tyrosine hydroxylase (TH) immunoreactivity was significantly elevated in the SNpc of Parkinsonian animals following ursolic acid therapy. It was observed that ursolic acid treatment reversed the neuroinflammation, neurodegeneration, and abnormalities in biochemical and behavioral markers [121]. 

**Figure 4:**
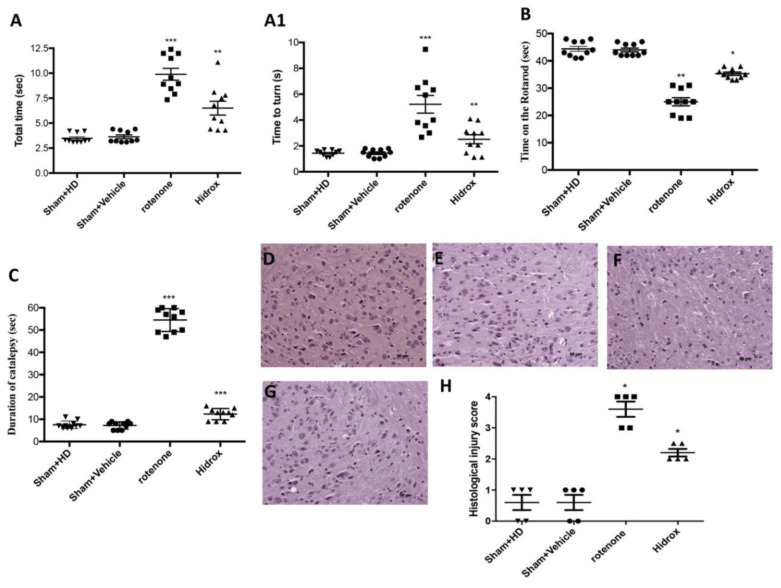
Effect of Hidrox^®^ on behavioral impairments and histological parameters induced by rotenone intoxication. (**A**,**A1**) Motor function was assessed using a Pole test. At 28 days, mice exhibited a significant motor dysfunction as indicated by an increase in “Time to turn” and “Total time” spent to descend to the floor following injection of rotenone compared with the Sham group. Hidrox^®^ administration notably reduced “Total time” and “Time to turn”. (**A**) *** *p* < 0.001 vs. Sham; ** *p* < 0.01 vs. rotenone; (**A1**) *** *p* < 0.001 vs. Sham; ** *p* < 0.01 vs. rotenone. (**B**) At 28 days, using a Rotarod apparatus, mice exhibited a significant motor dysfunction as indicated by a decrease in time spent on the Rotarod. Hidrox^®^ treatment blunted the motor dysfunction in mice. (**B**) ** *p* < 0.01 vs. Sham; * *p* < 0.05 vs. rotenone. (**C**) Catalepsy was evaluated according to the standard bar hanging procedure; this motor test showed that the Hidrox^®^ treatment reduced behavioral impairment induced by rotenone. (**C**) *** *p* < 0.001 vs. Sham; *** *p* < 0.001 vs. rotenone. Values are the mean ± SEM of 10 mice for each group. Sham+Hidrox^®^ and Sham+vehicle groups showed no evidence of degenerating cells in the SN (**D**,**E**), whereas degeneration of neuromelanin-pigmented cells was evident in the SN of the rotenone-treated animals (**F**). Hidrox^®^ treatment restored the architecture compared to the control mice (**G**). The data are representative of at least three independent experiments and are expressed as the mean ± SEM of 5 mice for each group. (**H**) * *p* < 0.05 vs. Sham; * *p* < 0.05 vs. rotenone. Scale bar: 50 μm. This figure is reprinted under the terms of the Creative Commons Attribution 4.0 International License from reference [122].

Similarly, in another study, the effectiveness of Hidrox^®^ was evaluated in a rotenone-induced PD mouse model [122]. Hidrox^®^ is an aqueous portion of olives extracted from defatted olive pulps. The major component of Hidrox^®^ is hydroxytyrosol, which is well-recognized for its antioxidant and anti-inflammatory properties. Following Hidrox^®^ treatment, various behavioral and neurological changes were evaluated in rotenone-induced PD mice. In the pole test, the “Time to turn” and “Total time” were significantly increased in the case of rotenone-treated mice compared with the Sham group (Figure 4A,A1). However, Hidrox^®^ treatment considerably decreased “Time to turn” (61%) and “Total time” suggesting a reduction in bradykinesia compared to control mice (Figure 4A,A1). The rotarod test revealed that Hidrox^®^-treated mice could significantly reduce motor deficits compared to the control animals (Figure 4B). Furthermore, the daily Hidrox^®^ treatment decreased rotenone-induced catalepsy duration (Figure 4C). Histopathological analysis of the brain discovered drastic changes via cytoplasmic vacuolization, vascular degeneration, and nigrostriatal neuronal cell loss in rotenone-treated mice compared to untreated mice (Figure 4D–H). However, Hidrox^®^-treated mice exhibited minimal vascular degeneration and cytoplasmic vacuolization.

### 4.3. ALS

Neuroinflammation is a crucial factor in ALS pathophysiology, contributing to the degeneration of motor neurons. The hallmarks of neuroinflammation in ALS are the activation of microglia and reactive astrocytes, lymphocyte and macrophage infiltration, and the overproduction of inflammatory cytokines [123]. Depending on the disease’s progression stage, immune cells may have protective or detrimental effects on motor neuron survival; however, the exact mechanism is unknown.

COX enzymes are involved in the biosynthesis of prostaglandins and thromboxane from arachidonic acid, which play an essential role in inflammation [124]. Therefore, it was thought that NSAIDs, which inhibit COX, could reduce the incidence of ALS and slow the progression of symptoms [125,126]. A meta-analysis was conducted to assess the relationship between the usage of acetaminophen (paracetamol), non-aspirin-NSAIDs (NA-NSAIDs), and aspirin and the risk of ALS [127]. It has been revealed that using NA-NSAIDs and acetaminophen reduces the likelihood of developing ALS, and these drugs appear to have neuroprotective properties. On the other hand, aspirin had no impact in lowering the chance of developing ALS.

A double-blind, placebo-controlled clinical trial was carried out to evaluate the effect of celecoxib treatment in ALS patients [128]. Three hundred ALS patients were randomized (2:1) to receive celecoxib (800 mg/day) or a placebo for a year. Individuals with ALS did not benefit from celecoxib therapy at the examined dose. However, celecoxib was well tolerated and did not cause an increase in the incidence of adverse events. Interestingly, prostaglandin E (2) levels in CSF were not raised at baseline and did not decrease after therapy.

**Figure 5:**
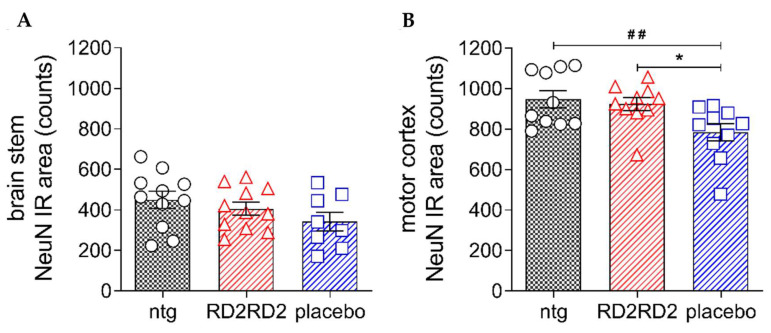
Treatment with RD2RD2 rescued significant numbers of neurons in the motor cortex of SOD1*G93A mice. Analysis of neurons in the brain stem (**A**) and motor cortex (**B**) revealed a significant loss in placebo-treated SOD1*G93A mice, while the count of neurons in RD2RD2-treated mice was similar to the count in non-transgenic mice. Data are presented as mean ± SEM. Statistical calculations were conducted by one-way ANOVA with Fisher’s LSD post hoc analysis, ntg *n* = 11, RD2RD2 *n* = 11, placebo *n* = 8 (brain stem) and ntg *n* = 10, RD2RD2 *n* = 10, placebo *n* = 10 (motor cortex). Lozenges and asterisks (*) indicate a significance between treatment groups (ntg vs. RD2RD2 or ntg vs. placebo: ## *p* = 0.01 and RD2RD2 vs. placebo: * *p* = 0.05). IR: immunoreactivity. Circles: placebo-treated ntg; triangles: RD2RD2-treated SOD1*G93A mice and squares: placebo-treated SOD1*G93A mice. This figure is reprinted under the terms of the Creative Commons Attribution 4.0 International License from reference [129].

In a study, Post et al. [129] examined the therapeutic potential of d-enantiomeric peptide RD2RD2 in the ALS SOD1*G93A transgenic mouse model. Compared to the placebo control, mice treated with RD2RD2 peptide had minimized activated microglia and astrocytes in the brainstem and lumbar spinal cord. Furthermore, peptide treatment was capable of rescuing neurons in the brain stem (Figure 5A) and motor cortex (Figure 5B). Although the target site is unknown, RD2RD2 treatment slowed disease phenotype progression during the treatment period.

Masitinib is a selective tyrosine kinase inhibitor that inhibits proinflammatory cytokines, reducing inflammation and enabling neuroprotection [130]. In a randomized clinical trial, masitinib showed significant benefits over a placebo in reducing the rate of functional decline in ALS patients [131]. Moreover, it exhibited a satisfactory safety profile and no severe toxicity on dosing daily at 4.5 or 3.0 mg/kg. 

### 4.4. HD 

Neuroinflammation is a common feature of NDs where infiltration of immune cells is observed in the brain [132]. Unlike other NDs, HD does not exhibit a considerable migration of peripheral immune cells into the brain [133,134]. Instead, activated microglia and astrocytes are responsible for most of the inflammatory processes in HD. While reactive T cells are absent from HD brains [135], reactive astrocytes are seen in presymptomatic HD and are correlated with the severity of the illness [136]. In post-mortem studies, neuron loss is noted in the striatum and cortex of HD brains, regions that are home to reactive microglia [134]. 

In a recent study, the neuroprotective effects of safranal or candesartan were studied in a 3-nitropropionic acid (3-NP)-induced HD rat model [137]. Treatment with 3-NP significantly reduced striatal neurotransmitter levels while increasing oxidative stress and inflammatory modulators, thereby decreasing memory and locomotor functions. Nevertheless, the co-administration of safranal or candesartan along with 3-NP could minimize or prevent behavioral, biochemical, and histological changes in the treated animals.

Studies have shown cannabinoids exert a neuroprotective role in multiple HD animal models like mitochondrial toxins treated rodents, human huntingtin mutated mice, and quinolinic acid-induced mice [138,139,140,141,142]. Besides anti-inflammatory activity, cannabinoids also exert neuroprotective potential through cannabinoid receptor type 1 (CB1) and type 2 (CB2) activation as well as antioxidant properties. In one study, the 3-NP-induced HD rats were treated with either arachidonyl-2-chloroethylamide (ACEA), HU-308, or cannabidiol (CBD). The ACEA is a CB1 agonist, while HU-308 is a CB2 agonist. In contrast, CBD possesses a minimum affinity for CB receptors. The treatment of 3-NP in rats leads to reduced gamma-aminobutyric acid (GABA) content and decreased levels of striatal GABAergic projection neuronal markers. The 3-NP treatment also reduced mRNA expression of SOD-1 and SOD-2, which are responsible for their antioxidant properties. However, treatment of CBD in an HD rat model with inverse 3NP induced reduction, but these effects were not observed in ACEA and HU-308 treated mice. Despite cannabinoid’s therapeutic potential against animal models, the clinical trial revealed no significant benefit [143]. However, a recent clinical trial has tested the use of cannabinoids to subdue HD symptoms like dystonia [144].

Instead of traditional anti-inflammatory agents, other anti-inflammatory drugs, such as minocycline, have been examined against HD. Kalonia et al. [145] examined the possible neuroprotective function of minocycline in the quinolinic acid-induced HD rat model. Quinolinic acid-treated rats exhibited reduced body weight, impaired motor function, and increased oxidative damage. Rats treated with minocycline at doses of 25, 50, and 100 mg/kg for 3 weeks showed improved motor function as shown by the rotarod test and balance beam walk performance. The treated rats also displayed increased body weight and improved oxidative defense. In a separate study, a combination of minocycline with coenzyme Q10 showed a reversing of different behavioral and neuropathological changes in the R6/2 mouse [146]. Moreover, the combination therapy showed considerably longer survival and enhanced motor function than minocycline or CoQ10 alone. Also, combined treatment diminished striatal neuron atrophy, gross brain atrophy, and huntingtin aggregation in the R6/2 mice compared to individual therapy. 

A brief list of anti-inflammatory drugs used in preclinical studies for the management of various NDs is given in Table 2.

## 5. Conclusions and Future Prospective

Despite extensive research, the complete understanding of the pathogenesis of NDs is still limited, creating hurdles for developing therapeutic agents. Most of the approved drugs for NDs are associated with symptomatic treatment instead of reversing the progression of NDs. Numerous emerging pieces of evidence have shown that inflammation not only promotes the course of NDs but can also act as an initiator. For instance, it was shown that inflammation manifests far sooner than protein aggregation in a PD mouse model. However, the relationship between neuroinflammation and NDs is dynamic and complex. Reactive microglia and astrocytes are common hallmarks of NDs. In a healthy brain, microglia and astrocytes play a critical role in neuroprotection. Meanwhile, activated microglia and astrocytes produce a proinflammatory milieu that exacerbates neuronal injury and disrupts the delicate balance of neurochemical signaling in the brain. 

There is potential for ND treatment by targeting neuroinflammation in multimodal approaches. Anti-inflammatory drugs have shown considerable benefits in numerous small animal models. Unfortunately, in clinical trials, anti-inflammatory medication fails to reverse or even slow down neurodegeneration. This difference might be partially attributed to limitations of the experimental animal model, since none fully phenocopies human NDs [164,165]. On the other hand, the therapeutic response of anti-inflammatory agents might depend upon ND phenotypes and stages of disease progression [166]. 

Therefore, it is questionable whether a single neuroinflammatory pathway can be chosen to manage various NDs. Instead, neuroinflammation and the corresponding targeting strategy should be selected depending on the ND type and its progression. Thus, a fully established mechanism of neuroinflammatory response in specific ND is required. Several medicines targeting distinct inflammatory signaling pathways are currently in different phases of clinical trials, which will most likely provide effective therapeutic strategies for patients with NDs in the future (Table 3).

In recent years, cell-based therapy has emerged as an alternative treatment strategy to ameliorate neuronal damage associated with various NDs. Among these, neural stem cells (NSCs) are regarded as one of the best options due to their ability to remediate nerve injury and nerve regeneration because of their capacity to differentiate into neurons, oligodendrocytes, and astrocytes [167]. According to reports, intravenous administration of NSCs significantly reduced the peripheral neuropathic pain caused by chronic constriction injuries of the sciatic nerve [168]. It has been shown that transplantation of NSCs into injured rat spinal cord segments significantly enhanced neurofilament protein expression while down-regulating the expression of glial fibrillary acidic protein and P2X4 and P2X7 receptors. Consequently, locomotor and sensory functions were considerably improved, which played an essential role in alleviating neuropathic pain after spinal cord injury [169]. Apart from this, mesenchymal stem cells (MSCs) have been extensively used due to their favorable characteristics, including ease of isolation from healthy donor and patient tissue, in vitro expansion, established biosafety profile, and lack of ethical concerns [170,171]. Co-administration of MSCs with resveratrol in the AD murine model enhanced MSCs’ engraftment in the hippocampus, improving neurogenesis, minimizing neuronal loss, and elevating learning and memory [172]. Despite amazing breakthroughs in stem cell research for neurodegenerative diseases, some crucial concerns remain to be addressed. The development of stem cell therapies is significantly impeded by the need to understand how stem cells function within the body and integrate with the intended tissue or organ.

## Figures and Tables

**Table 1:** List of FDA-approved medicines for managing various neurodegenerative disorders (NDs) with their dosage forms and common side effects.

Neurodegenerative Disorders	Drug Class	Drug Name	Dosage Form	Side Effects	References
AD	Cholinesterase inhibitors	Donepezil	Tablet	Nausea, vomiting, diarrhea, anorexia, weight loss, muscle cramps, insomnia, fatigue	[76,77,78]
		Rivastigmine	Capsule, transdermal patch	Nausea, vomiting, diarrhea, anorexia, weight loss, indigestion, fainting, muscle weakness	[76,78,79]
		Galantamine	Tablet, capsule, oral solution	Nausea, vomiting, diarrhea, decreased appetite, weight loss, headache, dizziness	[76,78]
	N-methyl-D-aspartate (NMDA) antagonist	Memantine	Tablet, capsule, oral solution	Diarrhea, dizziness, headache, constipation, confusion	[76,78]
	Atypical antipsychotic	Brexpiprazole	Tablet	Common cold symptoms, dizziness, shaking, increased appetite, weight gain, high blood sugar, stroke	[76]
	Disease-modifying immunotherapy	Lecanemab	Intravenous infusion	Brain swelling and bleeding, headache, confusion, dizziness, nausea, vomiting, cough, diarrhea, visual changes, body aches, fever, fatigue, slow or fast heartbeat, loss of appetite	[76]
		Aducanumab	Intravenous infusion	Brain swelling and bleeding, headache, confusion, dizziness, falls, diarrhea, visual changes	[76,78]
PD	Dopamine precursor with metabolic inhibitor	Levodopa/carbidopa	Tablet (IR, ER), capsule, enteral suspension, inhaler	Nausea, vomiting, low blood pressure, anorexia, confusion, dizziness, dyskinesia, nervousness, hallucinations	[78,80]
	Dopamine agonists	Pramipexole	Tablet	Dizziness, drowsiness, confusion, hallucinations, nausea, low blood pressure, leg swelling, sleep attacks, compulsive behaviors like gambling, vision problems, tiredness	[78,80]
		Ropinirole	Tablet	Dizziness, drowsiness, confusion, low blood pressure, leg swelling, nausea, vomiting, constipation, dry mouth, headache, tiredness, compulsive behaviors like gambling	[78,80]
		Apomorphine	Subcutaneous injection, sublingual film	Injection: Allergic reactions, dizziness, drowsiness, low blood pressure, leg swelling, confusion, sleep attacks, nausea, headache, compulsive behaviors like gambling Sublingual: nausea, mouth swelling, pain and sores, dizziness, sleepiness	[78,80]
		Rotigotine	Transdermal patch	Nausea, vomiting, dizziness, drowsiness, confusion, tiredness, headache, low blood pressure, skin rashes, sleep attacks, compulsive behaviors like gambling	[78,80]
		Bromocriptine	Tablet, capsule	Blurred vision, double vision, nausea, vomiting, low blood pressure, headache, dizziness, drowsiness, constipation, loss of appetite, tiredness	[81]
	Monoamine oxidase-B inhibitors	Rasagiline	Tablet	Nausea, dry mouth, dizziness, drowsiness, strange dreams, constipation, stomach/abdominal pain, heartburn, weight loss, may worsen dyskinesia	[78,80]
		Selegiline	Tablet, orally disintegrating tablet, capsule	Nausea, dry mouth, dizziness, constipation, abdominal pain, insomnia, headache, may worsen dyskinesia	[78,80]
		Safinamide	Tablet	Nausea, dry mouth, dizziness, constipation, insomnia, may worsen dyskinesia	[78,80]
	Catechol-O-methyltransferase inhibitors	Entacapone	Tablet	Diarrhea, discolored urine, potentiate levodopa related side effects, especially dyskinesia and confusion	[78,80]
		Tolcapone	Tablet	Diarrhea, discolored urine, enhanced side effects of levodopa especially dyskinesia	[78,80]
		Opicapone	Capsule	Dizziness, drowsiness, dyskinesia, weight loss, constipation, low blood pressure	[78,80]
	Anticholinergics	Benztropine	Tablet	Confusion, hallucinations, memory issues, blurred vision, dry mouth, nausea, constipation, flushing, urinary retention	[78,80]
		Trihexyphenidyl	Tablet, elixir	Confusion, hallucinations, memory issues, blurred vision, dry mouth, urinary retention	[78,80]
	Adenosine A2A antagonists	Istradefylline	Tablet	Dyskinesia, hallucinations, dizziness, insomnia, nausea, constipation	[78,80]
	N-methyl-D-aspartate (NMDA) antagonist	Amantadine	Tablet, capsule, syrup	Dizziness, hallucination, confusion, dry mouth, nausea, constipation, insomnia, urinary retention, peripheral edema	[78,80]
ALS	Glutamate-receptor antagonist	Riluzole	Tablet, oral suspension, oral film	Dizziness, drowsiness, weakness, nausea, vomiting, increased blood pressure, numbness/tingling around the mouth	[78]
	Free radical scavenger	Edaravone	Oral suspension, IV infusion	Bruising, gait disturbances, tiredness, headache	[78]
	Neuronal apoptosis inhibitors	AMX 0035 (Sodium phenylbutyrate and Taurursodiol)	Oral suspension	Diarrhea, nausea, abdominal pain, decreased appetite, upper-respiratory-tract infections, dizziness, drowsiness, tiredness	[82]
	Antisense oligonucleotide	Tofersen	Intrathecal injection	Joint pain, muscle pain, nerve pain, tiredness, or weakness	[83]
HD	Chorea medication	Tetrabenazine	Tablet	Drowsiness, dizziness, insomnia, depression, tiredness, nausea, vomiting	[84]
		Deutetrabenazine	Tablet	Drowsiness, dizziness, insomnia, depression, tiredness, dry mouth, nausea, vomiting	[85]
	Antipsychotic medication	Olanzapine	Tablet	Drowsiness, dizziness, headache, dry mouth, weight gain, constipation, stomach upset	[86]
		Risperidone	Tablet, oral solution	Drowsiness, dizziness, blurred vision, drooling, abdominal pain, dry mouth, nausea, weight gain, tiredness	[87]
	Antidepressants (selective serotonin reuptake inhibitors)	Citalopram	Tablet, capsule, oral solution	Nausea, drowsiness, dizziness, blurred vision, loss of appetite, dry mouth, tiredness, sweating, yawning	[88]
		Fluoxetine	Tablet, capsule, oral solution	Nausea, drowsiness, dizziness, anxiety, insomnia, loss of appetite, dry mouth, tiredness, sweating, yawning	[89]
		Sertraline	Tablet, capsule, oral solution	Nausea, drowsiness, dizziness, dry mouth, loss of appetite, diarrhea, upset stomach, insomnia, increased sweating	[90]
	Mood stabilizers	Lamotrigine	Tablet	Dizziness, drowsiness, headache, nausea, vomiting, upset stomach, sweating	[90]
		Carbamazepine	Tablet, capsule, oral suspension	Drowsiness, dizziness, nausea, vomiting, constipation, dry mouth, loss of appetite, skin rash, unsteadiness	[90]

**Table 2:** Drugs targeting neuroinflammation in various NDs.

Disease	Drug	Cellular/Animal Model	Dosage Regimen	Major Drug Target	Significant Outcomes	Reference
AD	Ibuprofen	Sprague Dawley rats	2 mg/kg/day intranasally for 6 days	Inactivation of *ras* homolog gene family member A (RhoA) to suppression of Aβ42 generation	Ibuprofen microemulsion displayed consistent colloidal dispersion characteristics, suitable drug release, and excellent stability. Intranasal delivery of ibuprofen microemulsion showed 4-fold higher brain delivery compared to the reference solution, and by approximately 4 and 10 times compared to intravenous and oral administration of ibuprofen microemulsion	[147]
	Curcumin	Streptozotocin-induced AD mice	5 mg/kg/day intranasally for 20 days	Inhibition of hyperphosphorylation of tau protein, reduction of the Aβ plaque formation, decreasing proinflammatory cytokines, and free radical scavenger.	Enabled intranasal curcumin delivery Reverted cognitive deficit in streptozotocin-induced AD mice	[148]
	ε-viniferin	In vitro: Murine primary neurons/astrocytes co-culture	1 μM pretreatment	Disaggregation of amyloid-β peptide.	Trans ε-viniferin promoted disaggregation of Aβ_42_. Alleviated inflammation in neuron/astrocyte co-cultures	[149]
	Naproxen	In vivo: AlCl_3_-induced AD rats	20 mg/kg/day intraperitoneally for 14 days	Inhibition of caspase-3 expression and also promotes neurogenesis.	Naproxen reduced AlCl_3_-induced neurocognitive impairment The combination treatment had no extra neuroprotective advantages over rivastigmine-only therapy except for promoting neurogenesis and suppressing apoptosis.	[150]
	4′-OH-flurbiprofen-chalcone hybrids	In vitro: BV-2 cell	0.5, 2.5, and 10.0 μM for 24 h	Inhibition of Aβ1–42 aggregation.	Inhibited self-induced Aβ1–42 aggregation and Cu^2+^-induced Aβ1–42 aggregation Exhibited good antioxidant activities and anti-neuroinflammatory properties Displayed appropriate blood-brain barrier permeability as determined by parallel artificial membrane permeation assay of the blood-brain barrier (PAMPA-BBB)	[151]
PD	Celecoxib	In vitro: SH-SY5Y cells	5, 15, 20, 25 and 50 μM for 6 h and 24 h	Inhibition of COX-2 enzymatic activity.	Celecoxib improves cell survival in 6-hydroxydopamine and paraquat in vitro models of PD. Promotes expression of the neuroprotective marker genes apolipoprotein D (*APOD*) and transcription factor EB (*TFEB*)	[152]
	Sodium salicylate	In vivo: Rotenone-treated SD rats	100 mg/kg/day intraperitoneally for 5 weeks	Inhibition of NF-κB, MAPK, or activator protein-1 (AP-1).	Sodium salicylate significantly improves dopamine and tyrosine hydroxylase levels as well as symptoms of motor dysfunction in rotenone-treated rats. After sodium salicylate co-treatment, levels of several inflammatory mediators decreased.	[153]
	Baicalin (5,6,7-Trihydroxyflavone)	α-syn/MPP^+^ and MPTP-induced PD mice	Pretreated with baicalin (0, 50, 100, 200 mg/kg/day) intragastrically for 7 days followed by a further administration for another 7 days during PD establishment.	Inhibition of NLRP3 inflammasome-mediated inflammation through the Nrf2 pathway.	Baicalin ameliorated MPTP and α-syn/MPP^+^ induced microglia activation, inflammatory response, and oxidative stress. Minimized dopaminergic neuron loss and motor dysfunction	[154]
	Rubusoside	MPTP-induced PD mice	150 mg/kg/day intragastric administration from day −3 (i.e., 3 days prior to MPTP administration) to day 24.	Inhibition of JNK/p38 MAPK/NF-κB signaling pathway.	Rubusoside inhibited microglial activation and thereby decreased the release of proinflammatory regulators. Improved motor function by preventing neuronal degeneration	[155]
	Benfotiamine	MPTP-induced PD rats	100 mg/kg/day (low dose) or 200 mg/kg/day (high dose) orally for 42 days	Targets multiple pathways, including suppression of NF-κB activation, prevents activation of p-38 MAPK and stress-activated kinases (SAPK/JNK), and NADPH-cytochrome c regulation.	Benfotiamine treatment improved MPTP-induced body balance, behavioral, and dopamine loss in the mid-brain. Ameliorated MPT-induced motor and nonmotor alterations	[156]
	Linagliptin	Rotenone-induced rat PD rats	5 mg/kg/day (low dose) or 10 mg/kg/day (high dose) orally for 28 days	DJ-1, Hypoxia-inducible factor 1-alpha (HIF-1α), potentiation in the Sirtuin 1 (SIRT-1)/Nuclear factor erythroid-2-related factor 2 (Nrf-2)/Heme oxygenase-1 (HO-1) pathway.	Linagliptin treatment (10 mg/kg) improved rotenone-induced motor dysfunction and histological injury. Inhibited inflammatory mediators, apoptosis, and oxidative stress	[157]
ALS	Nebivolol and donepezil	SOD1^G93A^ mice	Low dose (5 mg/kg/day nebivolol and 3 mg/kg/day donepezil orally) at 10 weeks of age for 70 days High dose: (9 mg/kg/day nebivolol and 6 mg/kg/day donepezil orally) from 63 days of age until the mice could not stand for 30 s	Combination therapy targets multiple key pathways, including inhibition of nuclear factor-κB (NF-kB) nucleus translocation, PI3K-dependent increase in neuronal viability under glutamate excitotoxicity, and upregulation of insulin growth factor-1 (IGF-1) mRNA.	Combination treatment deferred motor function decline and prevented motor neuronal degeneration in the spinal cord. Suppressed muscle atrophy and prolonged survival	[158]
	Masitinib	SOD1^G93A^ rats	30 mg/kg/day orally starting from day 1 or day 7 post-paralysis for 20 days or until end-stage	Inhibition of tyrosine kinase receptor colony-stimulating factor 1R (CSF-1R).	Masitinib treatment reduced microgliosis, the amount of aberrant glial cells, and motor neuron disease in the degenerating spinal cord in the SOD1G93A rats. Masitinib therapy started 7 days after paralysis onset increased post-paralysis survival by 40%.	[159]
	Fingolimod	SOD1^G93A^ mice	0.1 and 1 mg/kg intraperitoneally 3 times weekly from the onset of symptoms until the end of life	Sphingosine 1-phosphate (S1P) receptor modulator.	Fingolimod treatment significantly modulates neuroinflammatory and protective genes in ALS mice’s motor cortex and spinal cord. Improved neurological phenotype and prolonged survival of ALS mice	[160]
HD	Azilsartan	3-NP-induced HD rats	5 and 10 mg/kg/day orally for 14 days. Animals were euthanized on day 15.	Reduces angiotensin II type 1 receptors and NF-ĸB p65 expression.	Azilsartan treatment considerably ameliorated 3-NP-induced motor and behavioral dysfunction. Restored striatal GABA and glutamate levels in the treated rats	[161]
	Silyl resveratrol derivatives	3-NP-induced HD mice	20 mg/kg was administered intraperitoneally for 2 weeks (avoiding weekends) after disease onset (day 12 post-immunization), and data was collected for 7 weeks.	Like resveratrol, they modulate the AMPK/SIRT1/PGC-1α signaling network.	Di-triethylsilyl and di-triisopropylsilyl resveratrol derivatives showed superior anti-inflammatory and in vitro neuroprotective efficacy than resveratrol. 3,5-triethylsilyl-4′-(6″-octanoylglucopyranosyl) resveratrol significantly reduced HD progression in mice model.	[162]
	Mangiferin	3-NP-induced HD rats	10 and 20 mg/kg orally for 14 days	Inhibit IκB degradation and prevent NF-κB activation. It also acts as a free radical sequester.	Mangiferin treatment alleviated 3-NP-triggered anxiety, decreased motor function, reduced recognition memory, and lower neurological scoring. Attenuated 3-NP-induced histopathological changes in the brain hippocampus, cortex, and striatum sections	[163]

**Table 3:** Anti-inflammatory agents under clinical trials for different NDs.

Agent	Phase	Status	Sponsor	Conditions	CT.gov ID
Canakinumab	Phase 2	Active, not recruiting	Novartis Pharmaceuticals	Mild cognitive impairment or mild AD	NCT04795466
Celecoxib	Not applicable	Completed	University of California, Los Angeles	AD, dementia	NCT00065169
Rofecoxib and Naproxen	Phase 2 Phase 3	Completed	National Institute on Aging (NIA)	AD	NCT00004845
Indomethacin	Phase 3	Completed	Radboud University Medical Center	AD	NCT00432081
Prednisone	Phase 3	Completed	National Institute on Aging (NIA)	AD	NCT00000178
Lovostatin, Ibuprofen	Phase 4	Completed	National Institute of Mental Health (NIMH)	AD	NCT00046358
Rifaximin	Phase 1 Phase 2	Unknown	Taipei Medical University Shuang Ho Hospital	PD	NCT03958708
Ciprofloxacin/Celecoxib	Phase 2	Completed	NeuroSense Therapeutics Ltd.	ALS	NCT04165850
Anakinra	Phase 2	Unknown	Charite University, Berlin, Germany	ALS	NCT01277315
